# The Utrogestan and hMG protocol in patients with polycystic ovarian syndrome undergoing controlled ovarian hyperstimulation during IVF/ICSI treatments

**DOI:** 10.1097/MD.0000000000004193

**Published:** 2016-07-18

**Authors:** Xiuxian Zhu, Hongjuan Ye, Yonglun Fu

**Affiliations:** Department of Assisted Reproduction, Shanghai Ninth People's Hospital, Shanghai Jiaotong University School of Medicine, Shanghai, China.

**Keywords:** controlled ovarian stimulation, frozen-thawed embryo transfer, polycystic ovarian syndrome, premature LH surge, progesterone, Utrogestan

## Abstract

Poor oocyte quality is a main concern for decreased reproductive outcomes in women with polycystic ovarian syndrome (PCOS) during controlled ovarian hyperstimulation (COH). A primary way to improve oocyte quality is to optimize the COH protocol. It was demonstrated that the viable embryo rate per oocyte retrieved in the Utrogestan and hMG protocol, a novel regimen based on frozen-thawed embryo transfer (FET), is statistically higher than that in the short protocol. Thus, a retrospective study was conducted to evaluate the endocrine characteristics and clinical outcomes in PCOS patients subjected to the Utrogestan and hMG protocol compared with those subjected to the short protocol.

One hundred twenty three PCOS patients enrolled in the study group and were simultaneously administered Utrogestan and human menopausal gonadotropin (hMG) from cycle day 3 until the trigger day. When the dominant follicles matured, gonadotropin-releasing hormone agonist (GnRH-a) 0.1 mg was used as the trigger. A short protocol was applied in the control group including 77 PCOS women. Viable embryos were cryopreserved for later transfer in both groups. The primary outcome was the viable embryo rate per oocyte retrieved. The secondary outcomes included the number of oocytes retrieved, fertilization rate, and clinical pregnancy outcomes from FET cycles.

The pituitary luteinizing hormone (LH) level was suppressed in most patients; however, the LH level in 13 women, whose basic LH level was more than 10 IU/L, surpassed 10 IU/L on menstruation cycle day (MC)_9–11_ and decreased subsequently. No significant between-group differences were observed in the number of oocytes retrieved (13.27 ± 7.46 vs 13.1 ± 7.98), number of viable embryos (5.57 ± 3.27 vs 5 ± 2.79), mature oocyte rate (90.14 ± 11.81% vs 93.02 ± 8.95%), and cleavage rate (97.69 ± 6.22% vs 95.89 ± 9.57%). The fertilization rate (76.11 ± 19.04% vs 69.34 ± 21.81%; *P* < 0.05), viable embryo rate per oocyte retrieved (39.85% vs 34.68%; *P* < 0.05), biochemical pregnancy rate (71.72% vs 56.67%; *P* < 0.05), clinical pregnancy rate (64.65% vs 51.65%; *P* < 0.05), and implantation rate (46.46% vs 31.35%; *P* < 0.05) in the study group were significant higher than those in the control group.

This study shows that the Utrogestan and hMG protocol was feasible to improve the oocyte quality, possibly providing a new choice for PCOS patients undergoing IVF/ICSI treatments in combination with embryo cryopreservation.

## 1 Introduction

Polycystic ovarian syndrome (PCOS), defined by an ovulation/oligoovulation, irregular menses, and androgen excess through 2003 Rotterdam criteria, is a common endocrine disorder.^[1]^ The prevalence of PCOS in women of reproductive age varies between 6.3% and 21.4% depending on the population, and it is 5.6% in China.^[2,3]^ In vitro fertilization (IVF) is an important therapy for PCOS women, with controlled ovarian hyperstimulation (COH) being a key component.^[4,5]^ Although more oocytes were retrieved in PCOS patients than in normal women, poor oocyte quality, low fertilization rates, and high miscarriage rates were still a principal problem in PCOS patients undergoing IVF.^[6–9]^ A primary measure taken is the optimization of the COH protocols. Although various protocols have been used,^[4,5]^ the clinical outcomes of PCOS patients remain unsatisfactory.^[10]^ Therefore, new methods to improve clinical outcomes are still urgently needed.

Recently, progesterone soft capsules (brand name: Utrogestan) were demonstrated to be an effective oral alternative to prevent premature LH surges in normal ovulatory women,^[11,12]^ establishing a convenient user regimen in combination with frozen-thawed embryo transfer (FET). Additionally, the viable embryo rate per oocyte retrieved in the Utrogestan and hMG protocol was found to be higher than that in the short protocol. Thus, we conducted a retrospective study to evaluate the endocrine characteristics and clinical outcomes in PCOS patients undergoing COH using the Utrogestan and hMG protocol compared with the short protocol.

## 2 Materials and methods

### 2.1 Study setting and patients

This retrospective study was performed at the assisted reproduction clinic of a tertiary research and education hospital. Women undergoing IVF/intracytoplasmic sperm injection (ICSI) regimens for infertility treatment were recruited from April 2013 to April 2015. The protocol was approved by the Ethics Committee (Institutional Review Board) of the Ninth People's Hospital of Shanghai. Written informed consent was obtained from all of the patients.

The study group consisted of patients who were not older than 38 years of age diagnosed with PCOS according to the Rotterdam criteria.^[1]^ Patients with documented ovarian failure, endometriosis grade 3 or higher, any contraindications to ovarian stimulation treatment, or documented cycles with no oocyte retrieved were excluded.

### 2.2 Procedures

#### 2.2.1 Controlled ovarian stimulation and allocation

The Utrogestan and hMG protocol applied in the study group was according to the method described earlier.^[11,12]^ Briefly, the human menopausal gonadotropin (hMG) 150 to 225 IU (Maanshan Pharmaceutical Trading Co., Anhui, China) and Utrogestan (Laboratories Besins International, Paris, France) 100 mg twice a day from MC_3_ was administered until the trigger day. Follicular monitoring started at MC_9–11_ and was performed every 2 to 4 days; meanwhile, serum FSH, LH, E2, and progesterone concentrations were measured. The final stage of oocyte maturation was triggered using triptorelin 0.1 mg (Decapeptyl; Ferring Pharmaceuticals, Germany). A short protocol was used for the control group. Patients were administered 0.1 mg of triptorelin daily beginning on MC_2_ and 150 to 225 IU of hMG daily beginning on MC_3_. The initiating dose of hMG administration was based on the same criteria as for the study group. After 6 to 7 days, ultrasound examination and serum hormone level tests were performed and the dose of hMG was adjusted according to follicle development. When dominant follicles reached 18 mm in diameter, HCG (Lizhu Pharmaceutical Trading Co., Zhuhai, China) 3000 IU was injected for the final stage of oocyte maturation. Transvaginal ultrasound-guided oocyte retrieval was conducted 34 to 36 h after the trigger. All of the follicles with diameters greater than 10 mm were retrieved. The fertilization of the aspirated oocytes was performed by either IVF or intracytoplasmic sperm injection (ICSI), depending on the semen parameters. According to the number and regularity of blastomeres and degree of embryonic fragmentation, good-quality embryos (including grade 1 and grade 2, 8-cell embryos) were frozen by vitrification on the third day following oocyte retrieval, and non-top-quality embryos were placed in extended culture, of which good-morphological grade blastocysts were frozen on day 5 or day 6.

#### 2.2.2 Endometrium preparation and FET

In this study, the method of endometrium preparation was similar in both groups. Specifically, letrozole and, if necessary, hMG was employed to stimulate monofollicular growth. Letrozole 5 mg was administered from cycle day 3 to 7, and then follicle growth was monitored beginning on day 10. At times, the treatment included a low dose of hMG (75 IU/day) to stimulate follicular and endometrial lining growth. The administration of 5000 IU of hCG and timing of FET were performed as described elsewhere.^[11]^ The transfer of day 3 embryos or blastocysts was scheduled based on the embryo and endometrium synchronization. When pregnancy was achieved, the progesterone supplement was continued until 10 weeks of gestation.

Hormone replacement therapy was recommended for patients with a thin endometrium during either natural cycles or stimulation cycles. Oral ethinyl estradiol (EE; Shanghai Xinyi Pharmaceutical) 75 mg/day was administered from day 3 to attain the criteria of endometrial thickness ≥8 mm and a triple line pattern on ultrasound scans. At that time, the patients were given 0.4 g of progestin (Laboratoires Besins-Iscovesco) intravaginally daily, and embryo transfer was performed 3 days later under abdominal ultrasound guidance. Oral estradiol and progestin were continued until 10 weeks of gestation when pregnant.

### 2.3 Statistical analysis

The primary outcome measure was the viable embryo rate per oocyte retrieved. The secondary measures included the number of oocytes retrieved, fertilization rate, and clinical pregnancy outcomes from frozen-thawed embryo transfer (FET) cycles. The viable embryo rate per oocyte was defined as the number of viable embryos divided by the number of oocytes retrieved. Clinical pregnancy was defined as the presence of a gestational sac with fetal heart activity during ultrasound examination 7 weeks after FET. The implantation rate was defined as the number of gestational sacs divided by the number of embryos transferred. The miscarriage rate was defined as the proportion of patients with spontaneous termination of pregnancy. The cycle cancellation referred to the patients who completed the oocyte retrieval without viable embryos.

In the table presented in this study, the data are presented as the mean ± SD. The data were analyzed using one-way analysis of variance, using the Bonferroni method and Dunnett test as appropriate, and *P* < 0.05 was considered to be statistically significant. All of the data were analyzed using the Statistical Package for the Social Sciences for Windows software (version 16.0, SPSS Inc.).

## 3 Results

### 3.1 Patient characteristics

**Figure 1:**
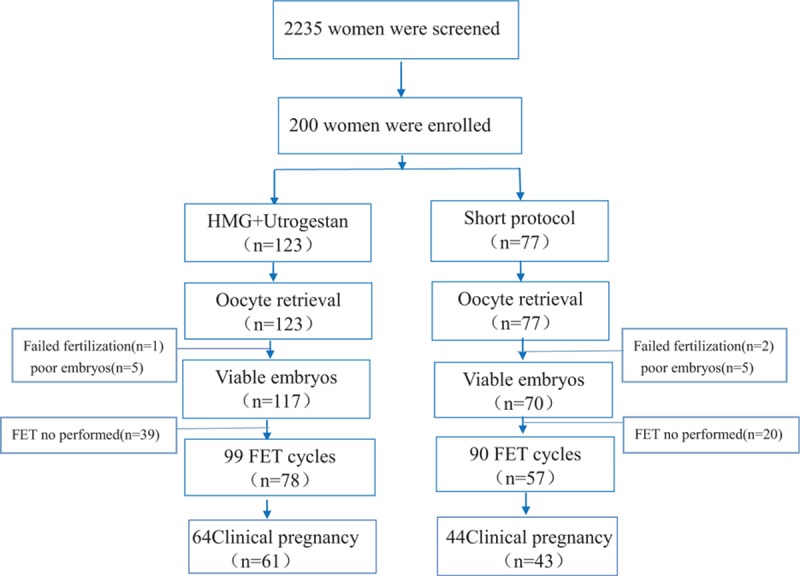
Flowchart of the study.

Figure 1 shows a profile summary of the study. In total, 2235 women were screened for the study, including 123 women in the study group (Utrogestan and hMG protocol) and 77 women in the control group (a short protocol). Two hundred women completed oocyte retrieval cycles, and 135 women completed FET cycles. All of the participants succeeded in producing oocytes (range, 1–57), and 187 women (93.5%) had the highest quality embryos to cryopreserve, while 13 patients were excluded from the study because they did not produce the highest quality embryos.

There were no significant between-group differences among the two groups in terms of age, BMI, number of antral follicles, duration of infertility, basal endocrine characteristics, and the proportion of indications (*P* > 0.05). In the study, 17.89% (20/123) of the study group patients and 20.78% (16/77) of the control group participants had previously failed FET treatments (*P* > 0.05) (Table 1).

**Table 1:**
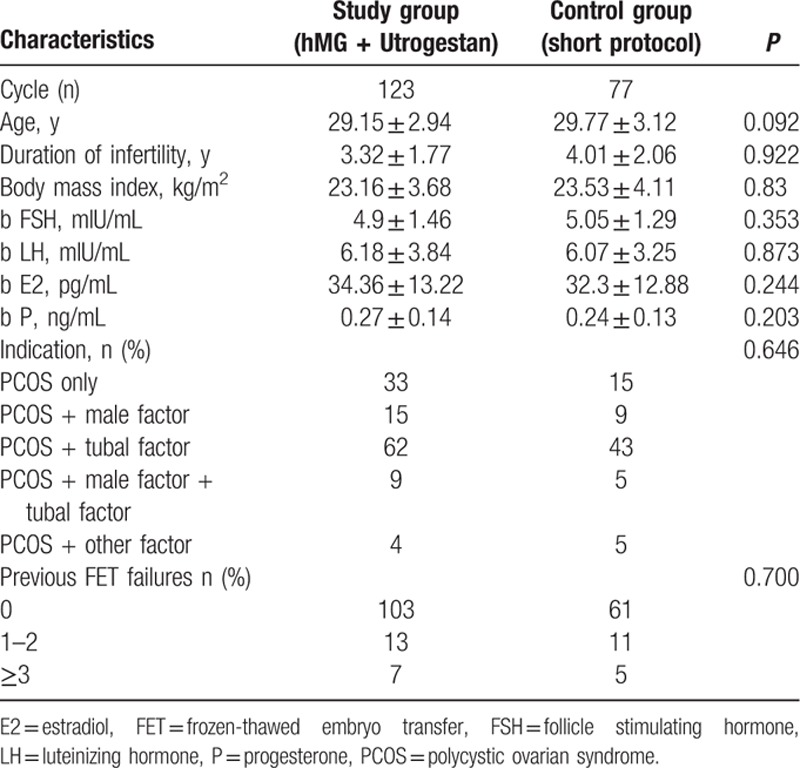
General patient information  
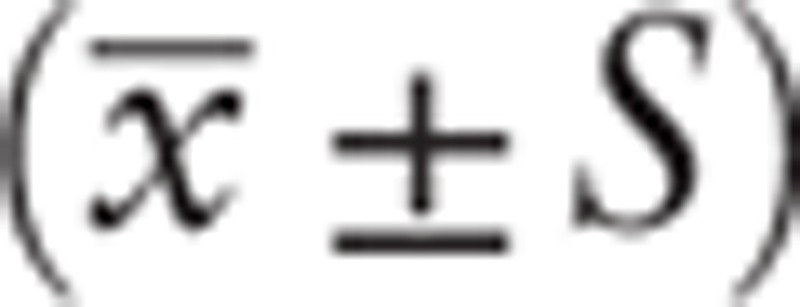
.

### 3.2 Hormonal profile

**Figure 2:**
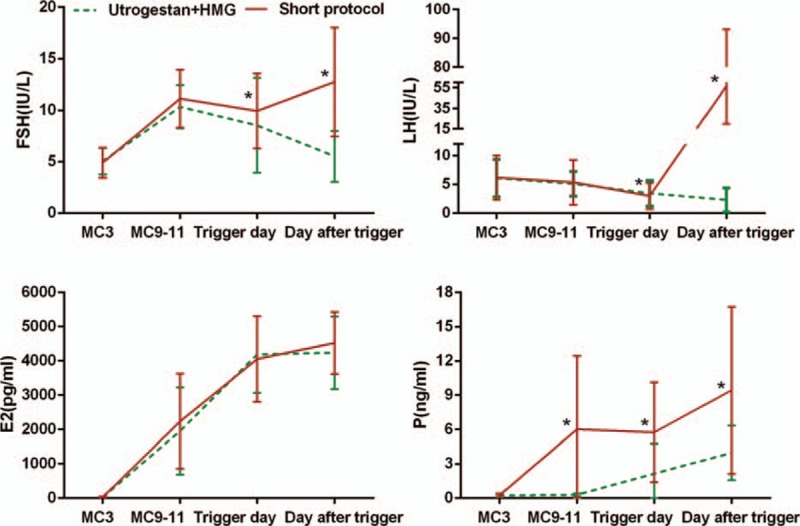
Serum hormone profiles present during ovarian stimulation in the 2 regimens. The red line refers to the Utrogestan + hMG group and the green line stands for the short-protocol group. The asterisk (∗) represents *P* < 0.05 at the time point.

Serum FSH, LH, E2, progesterone, and testerone in the 2 groups are shown in Fig. 2. In the study group, the FSH levels increased significantly after hMG administration and remained steady during ovarian stimulation. After trigger by GnRH-a, FSH increased to 12.75 ± 5.28 IU/L. The LH values gradually decreased during ovarian stimulation, which was 5.36 ± 3.8 IU/L on MC9–11 and 2.98 ± 2.25 IU/L on the trigger day, and increased significantly to 56.53 ± 36.54 IU/L 10 h later after trigger by GnRH-a. The serum E2 values increased gradually with the growth of follicles during ovarian stimulation. The serum P values increased after the delivery of Utrogestan, with a range of 0.9 to 43 ng/mL and were maintained at a stable concentration.

In the control group, FSH increased after hMG administration and then decreased slowly. The LH level decreased slowly, and the E2 value increased gradually. Meanwhile, the P value did not increase until the trigger day. Thus, the E2 value remained comparable between the two groups, the FSH and LH level showed statistic significance both in the trigger day and the day after trigger, and statistic differences were found in the P value since the delivery of Utrogestan.

### 3.3 Ovarian stimulation, follicle development, and oocyte performance

Table 2 describes the clinical and cycle characteristics of COH in both groups. No significant between-group differences were found in the mean stimulation duration and doses of hMG, number of follicles with a diameter larger than 10 and 14 mm, number of oocytes retrieved, MII oocytes, fertilized oocytes, cleaved embryos, D3 top-quality embryos and rate of oocyte retrieval, oocyte maturation, and cleavage (*P* > 0.05). The fertilization rate (76.11 ± 19.04% vs 69.34 ± 21.81%; *P* < 0.05) and viable embryo rate per oocyte retrieved (39.85% vs 34.68%; *P* < 0.05) were significantly higher in the study group than in the control group. The cycle cancellation rate due to the lack of viable embryos did not differ between the 2 groups (6.5% vs 9.1%; *P* > 0.05). Two patients experienced moderate or severe OHSS in each group during the study, respectively (*P* > 0.05).

**Table 2:**
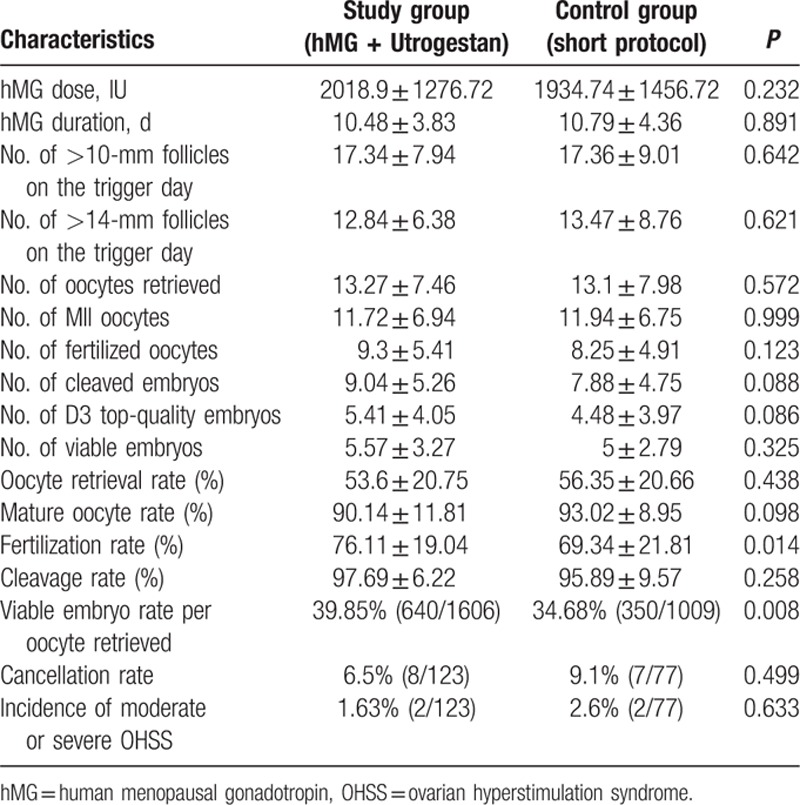
Stimulation and embryological characteristics of the patients  
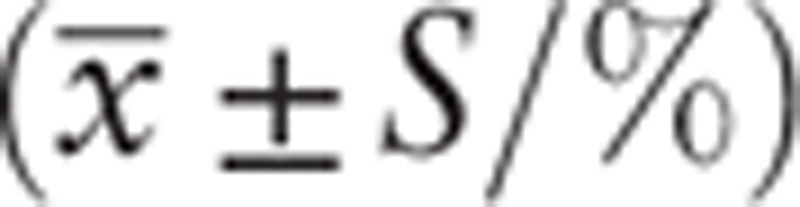
.

There were 13 of 123 cases presenting LH levels more than 10 IU/L on MC_9–11_ (13.62 ± 4.11 IU/L), which decreased to below 10 IU/L subsequently (5.62 ± 2.78 IU/L). The progesterone value in these patients was 3.85 ± 2.67 IU/L on MC_9–11_ and increased to 5.08 ± 2.49 IU/L on the trigger day. The number of oocytes retrieved was 12.08 ± 5.28, and the mature oocyte rate was 95.85 ± 5.91% in these patients, with no significant differences compared with others (*P* > 0.05). However, the number of viable embryos (3.54 ± 3.07) was less than the average level (*P* < 0.05).

### 3.4 Pregnancy

In our study, 135 women completed a total of 189 FET cycles, including 98 women who underwent 1 FET, 22 women who completed 2 FETs, 13 women who finished 3 FETs, and 2 women who completed 4 FET cycles. The remaining 65 women did not complete their FET cycles for personal reasons before the end of the study (Table 3).

**Table 3:**
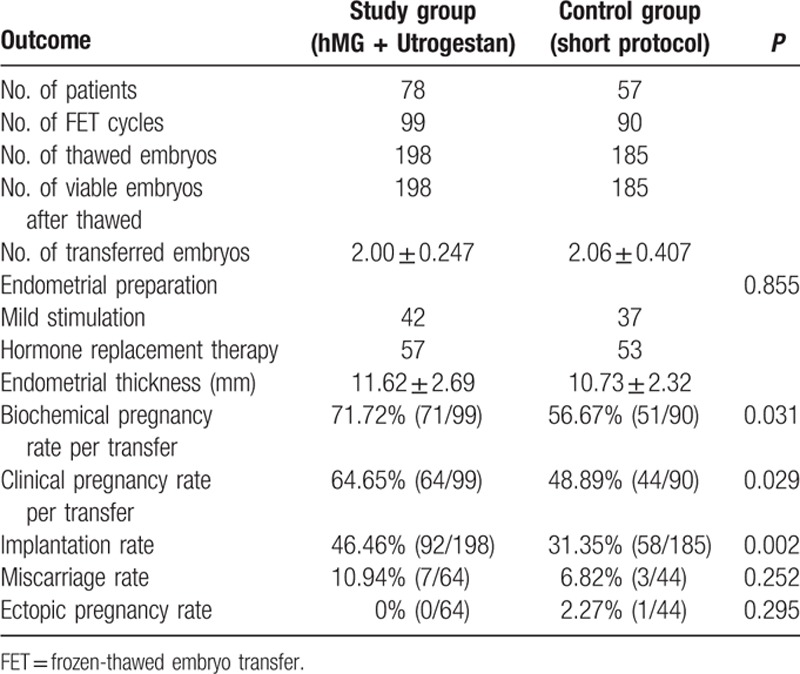
Pregnancy outcomes of frozen-thawed embryos originating from the two regimens.

In total, 383 embryos were thawed, and the rate of viable frozen-thawed embryos was 100% (383/383). No between-group difference was found in the endometrial preparation. The biochemical pregnancy rate (71.72% vs 56.67%), clinical pregnancy rate per transfer (64.65% vs 48.89%), and implantation rate (46.46% vs 31.35%) in the study group was higher in the study group than in the short-protocol group (*P* < 0.05), indicating that the embryos in the study group shared better development potential those in the control group. Notably, 10.94% (7/64) of the patients in the study group had a miscarriage before reaching the gestational age of 12 weeks, while 6.82% (3/44) of patients in the short-protocol group miscarried.

Of all the pregnancies in the study group, 22 women had live births, and 35 had ongoing pregnancies in the study group compared with 36 live births and 5 ongoing pregnancies in the control group by the end of the research period. Delivery follow-up showed 8 twin births and 14 single births in the Utrogestan and hMG protocol in contrast to 11 twin births and 25 single births. All of the newborns were healthy during follow-up.

## 4 Discussion

The LH level was suppressed after a 6-day Utrogestan treatment, and no premature LH surge was observed during COH in normal-ovulatory women.^[11,12]^ In PCOS patients, the pituitary LH levels were suppressed in most patients; however, in 13 women, it surpassed 10 IU/L on MC_9–11_ and decreased subsequently. Generally, the range of the LH level in the follicular phase was 5 to 10 IU/L in the natural menstrual cycle.^[13]^ Presently, most of the definitions of premature LH surge were based on the levels of LH and P. The cutoff level of premature LH surge was 10 IU/L, 12.4 IU/L, or twice more than the basic LH level,^[14–17]^ while the threshold level of progesterone varied from 1 ng/mL to 2.25 ng/mL.^[18–20]^ There was no consensus with respect to the definition of the premature LH surge in the GnRH-a, GnRH antagonist and mild stimulation protocol, let alone the Utrogestan and hMG protocol.

It was well known that the premature LH surge can compromise the oocyte yield and reduce pregnancy rates.^[21]^ A study concerning patients undergoing COH with a GnRH antagonist who experienced a transient premature rise in LH observed equivalent clinical pregnancy rates in these patients compared with those in normal women.^[22]^ Parallel to this finding, patients with a consistently high LH level (>10 IU/L) was demonstrated to obtain high-quality embryos in PCOS women undergoing minimal stimulation, reinforcing the notion that a consistent high LH level may not exert a negative role in the embryo quality, and 10 IU/L was not a good indicator for premature LH surge.^[13]^ In line with these findings, the mature oocyte rate in these patients with a higher LH level on MC_9–11_ was not impaired in our study, although there is a decrease in the number of viable embryos. However, the results should be evaluated with caution as a limited number of cases were present, which is a major limitation of this study. Additional research should be performed to determine the impact of altered LH levels on oocytes and embryo quality and whether 10 IU/L is a valuable cut-off level for predicting premature LH surge in PCOS patients undergoing the Utrogestan and hMG protocol.

The reason why the LH level failed to decrease timely after taking the same dose of Utrogestan orally can be elucidated from the following aspects. First and foremost, a clear dose–time effect has been demonstrated about the delivery of Utrogestan. A dose of 300 mg/d for 10 days could initiate bleeding in patients with secondary amenorrhea, while 25 days were needed for a dose of 100 mg/d.^[23]^ The lower progesterone level after taking the same dose of Utrogestan, as well as the high basic LH level that was more than 10 IU/L, may be one of the causes of the delayed onset time of Utrogestan in these women. In the future, we perhaps could prolong the interval between the application of Utrogestan and hMG in terms of the concentration of LH. In addition, there were certain mutations in LH, the LH receptor (LHR) or LH-regulated genes in some PCOS women that may change the biological activity of LH, leading to higher immunoactive LH levels needed to maintain normal follicle development.^[24–26]^

The fertilization rate, viable embryo rate per oocyte retrieved, biochemical pregnancy rate, clinical pregnancy rate, and implantation rate in the study group were higher than those in the control group. And there was statistic significance in the P level between the two groups since the delivery of Utrogestan, as shown in Fig. 2. Thus, one explanation is that the addition of Utrogestan benefited PCOS patients via altering the progesterone deficiency state. It was ascertained that the conversion of progesterone to androstenedione accelerated in theca cells of PCOS ovaries, leading to progesterone scarcity in PCOS women.^[27–29]^ Progesterone deficiency was prone to facilitate LH secretion in PCOS patients, as reported by Fiad et al,^[30]^ resulting in various disturbances. Additionally, progesterone played a crucial role, both directly and indirectly, in oocyte maturation, fertilization, and embryo development, and the effect was dependent on the progesterone concentration, as described in some previous articles.^[31–36]^ Thereby, the Utrogestan and hMG protocol is promising in the treatment of PCOS women, and the optimal dose of Utrogestan remains to be determined to warrant follicle development in an appropriate intrafollicular steroidogenic milieu.

Ovarian hyperstimulation syndrome (OHSS) is a serious complication of ovarian stimulation that affects 1% to 14% of all IVF cycles.^[37,38]^ The incidence of moderate or severe OHSS (%) in the Utrogestan group (1.63%) was lower than that in the short-protocol group (2.6%), with no significant difference. Various prevention strategies applied in our clinic contributed to the low incidence of OHSS both in Utrogestan group and the short-protocol group, including the application of low-dose hCG in the late-follicular phase,^[39]^ vaginal delivery of the dopamine agonist cabergoline,^[37]^ trigger with low-dose hCG,^[40]^ and “freeze-all" strategy.^[37]^

A major limitation of our study is the retrospective design of this study. Additionally, the number of participants enrolled was insufficient; thus, the number of women with LH >10 IU/L on MC9–11 was limited, decreasing the power of this study. On the other hand, the exact number of antral follicles was not provided as 20+ or PCO was used to record the number of antral follicles in PCOS patients in our clinic, one detail, which reduced accuracy. Finally, patients with short protocol did not need to take the hormone determination every time they come to the hospital in our clinic, because the physician was so familiar with it as a routine regimen. Thus, the hormone data of the short protocol was obtained only in part of the population enrolled.

To conclude, this study shows that PCOS women using the Utrogestan and hMG protocol undergoing IVF/ICSI treatments could obtain competent oocytes/embryos and optimal pregnancy outcomes in FET cycles. Many problems concerning this novel regimen in PCOS women remain to be explored such as the proper delivery time and dose of Utrogestan, and practicality and individualization of this new protocol. Therefore, more trials should be implemented using large samples. Moreover, basic studies are indispensable to seek the alterations in extra- and intra-ovarian factors in PCOS patients using the Utrogestan and hMG protocol and to help understand the action mechanism of Utrogestan, as well as to optimize this novel protocol in individuals.
